# Toxic, Oviposition Deterrent and Oxidative Stress Effects of *Thymus vulgaris* Essential Oil against *Acanthoscelides obtectus*

**DOI:** 10.3390/insects11090563

**Published:** 2020-08-24

**Authors:** Jelica Lazarević, Stojan Jevremović, Igor Kostić, Miroslav Kostić, Ana Vuleta, Sanja Manitašević Jovanović, Darka Šešlija Jovanović

**Affiliations:** 1 Institute for Biological Research “Siniša Stanković”—National Institute of Republic of Serbia, University of Belgrade, Bulevar Despota Stefana 142, 11060 Belgrade, Serbia; ana.vuleta@ibiss.bg.ac.rs (A.V.); manitas@ibiss.bg.ac.rs (S.M.J.); darka.seslija@ibiss.bg.ac.rs (D.Š.J.); 2 Bayer d.o.o., Omladinskih brigada 88b, 11070 Belgrade, Serbia; stojan.jevremovic@bayer.com; 3 Institute for Multidisciplinary Research, University of Belgrade, Kneza Višeslava 1, 11030 Belgrade, Serbia; igork@imsi.bg.ac.rs; 4 Institute for Medicinal Plant Research “Dr Josif Pančić”, Tadeuša Košćuška 1, 11000 Belgrade, Serbia; mkostic@mocbilja.rs

**Keywords:** *Acanthoscelides obtectus*, thyme essential oil, residual contact toxicity, progeny production, oviposition deterrent index, oxidative damage, thiols

## Abstract

**Simple Summary:**

Compounds of botanical origin, including essential oils (EOs), which coevolved as plant defense mechanism against herbivores and pathogens have been proposed as a promising strategy for post-harvest control of stored product insects. Despite several drawbacks, such as low stability, phytotoxicity and food odor changes at high concentrations, EOs are believed to be safe for human and environmental health and thus have an advantage comparing to conventional insecticides. The present study was aimed to examine acute toxicity and effects of thyme (*Thymus vulgaris*) EO on longevity, behavior and physiology of the bean weevil (*Acanthoscelides obtectus*), a major pest of stored bean seeds. We found that exposure to thyme oil reduced adult survival and longevity and induced damages to lipids and proteins in a concentration-dependent manner. Sublethal EO concentrations also deterred female egg laying and strongly inhibited adult emergence. Efficacy of such low EO concentrations in suppression of adult emergence implies that thyme EO might be cost-effective and environmentally low risk botanical insecticide for bean seed protection against *A. obtectus*.

**Abstract:**

The bean weevil *Acanthoscelides obtectus* Say (Coleoptera: Chrysomelidae: Bruchinae) can cause significant losses in production of its primary host common bean *Phaseolus vulgaris* L. To avoid bean protection with environmentally risky chemical insecticides and provide sustainable and safe production of food, new pest management methods based on natural compounds are investigated. In the present study, we evaluated protective potential of the essential oil (EO) from the common thyme *Thymus vulgaris* L. applied on bean seeds. We assessed residual contact toxicity of thyme EO and its effects on *A. obtectus* longevity, oviposition and adult emergence. Furthermore, to elucidate the role of oxidative stress in thyme EO toxicity, we estimated the levels of oxidatively damaged proteins and lipids, as well as the level of thiols which have important role for antioxidant capacity. We found that thyme oil significantly reduced adult survival and longevity, induced oxidative damage to lipids and proteins and depleted protein and non-protein thiols in a concentration-dependent manner. Females appeared to be more tolerant to thyme oil treatment than males. Sublethal EO concentrations affected oxidative stress indices, deterred oviposition and strongly inhibited adult emergence. The results suggest that thyme oil has the potential to be used as an ecofriendly insecticide for *A. obtectus* control.

## 1. Introduction

Bean and other legume seeds are an important source of proteins in human nutrition [1]. Insect attacks account for the majority of losses in the production and storage [2]. Post-harvest losses include both quantitative and qualitative damages, i.e., both seed weight and nutritional value are decreased [3]. The bean weevil (*Acanthoscelides obtectus* Say), one of the most important pests of common bean, causes about 10% weight loss of bean seeds after one generation [4,5]. After 3–4 months of storage unprotected beans can be completely lost [6].

Fumigation of storages by chemical insecticides efficiently eliminates pests but bears many drawbacks regarding evolution of resistant insects, toxicity to humans and environment pollution [7]. Sustainable and safe production of food is gaining importance, especially due to alarming growth of the human population [8]. Compounds of botanical origin which coevolved as defense mechanisms against herbivores and pathogens have been proposed as a promising strategy but few are commercially produced [9,10]. Numerous investigations of their protective power against storage pests is taking place, and insecticidal and repellent properties of plant powders [11,12,13], plant extracts [14,15,16], and essential oils are tested [17,18].

Essential oils (EO) are mixtures of phenylpropanoids and terpenoids produced in secondary metabolism of the aromatic plants [8,18]. It is expected that their complex composition with many diverse compounds having multiple mode of action might slow down evolution of pest resistance. EOs rarely exhibit toxicity to mammals and, because of low persistence, are considered safe for the environment [19,20]. However, using EOs in pest control has several drawbacks. At higher concentrations EOs can be phytotoxic and also can change food odor [21]. Effective EO concentration could be reduced by synergistic combinations with other ecofriendly means of pest control [22,23,24,25,26]. On the other hand, drawbacks such as low stability, lower efficacy compared to chemical insecticides and the need for several reapplications can be overcome by improved delivery methods based on nanotechnology [8,10,27,28,29]. Although the biorationals like EOs are frequently believed to be safe, supposedly because of their natural origin, they can show various negative impacts that need to be investigated and highlighted [30]. Additionally, environmental risks of more toxic and persistent nanopesticides should be examined before their application [28].

Significant bioactivity against pest insects has been recorded for essential oils from 1500 plant species [31]. Many studies on storage pests have shown that contact and fumigant applications of EOs increase mortality, reduce adult emergence and provoke repellent, feeding and oviposition deterrent behavioral responses [32,33,34,35,36,37,38,39,40,41,42]. Negative effects on longevity and fecundity of pests as well as life history traits of their offspring have been recorded at sublethal concentrations [43,44,45,46]. However, physiological mechanisms underlying EO activity are poorly understood [8].

Direct toxicity of EOs is a consequence of neurotoxic action through acetylcholine esterase inhibition or interference with receptors of octopamine and GABA neurotransmitters, while indirect toxicity is related to numerous biochemical and physiological targets [47]. EOs may act as insect growth regulators [48,49], disrupt gut epithelium structure [50] or inhibit digestive [51] and detoxification enzymes [52,53,54,55]. At the cellular level, essential oils and their components may exert prooxidant effects by changes in electron flow through mitochondrial complexes and increasing reactive oxygen species (ROS) production which cause damages to macromolecules and apoptosis [56]. For example, prooxidant effects of EO from *Eugenia uniflora* (L.) leaves on *Drosophila melanogaster* were confirmed by elevation in ROS and lipid peroxides levels, as well as by activation of enzymatic antioxidants and proteins involved in stress response and antioxidant signaling [57].

Previous studies of EO effects in *A. obtectus* have involved EOs isolated from plants belonging to families of Amaranthaceae [58], Asteraceae [59], Cupressaceae [60], Lamiaceae [61,62,63], Lauraceae [64], Meliaceae [65], Myrtaceae [60,62,64,66], Piperaceae [65], Poaceae [67], Rutaceae [68]. In the present study we examined how thyme EO (*Thymus vulgaris* L., Lamiaceae, thymol chemotype) applied by contact affected *A. obtectus* mortality, F1 progeny production and choice of seeds for oviposition. Early works of Regnault-Roger and Hamraoui [69,70] showed that fumigant application of thyme oil and its dominant component thymol negatively affects survival and longevity of females and males, number of laid eggs, larval penetration into seeds, preadult survival and number of emerged adults. We have previously shown that contact application of thyme EO might protect bean seeds as well. Namely, screening for contact toxicity and antioviposition activity against *A. obtectus* revealed that thyme EO was more efficient than other tested Lamiaceae EOs (basil and rosemary EO) [71]. Here we determined effective concentrations which halved adult survival and adult emergence, and concentration which led to three-fold lower oviposition on treated than on control bean seeds in a two-choice test. The aim of the present work was also to elucidate the role of oxidative stress in toxicity of thyme EO. To achieve this goal, we determined the level of thiol groups which have an important role in antioxidant capacity as well as the level of malondialdehydes and carbonyls which are indicators of oxidatively damaged lipids and proteins, respectively.

## 2. Materials and Methods

### 2.1. Insects and Rearing Conditions

Bean weevils (*A. obtectus*) used in this study originate from the laboratory population maintained on bean seeds for more than 250 generations. In our experiments, bean weevils were reared at 27 ± 1 °C, 55 ± 10% relative humidity and 12 h:12 h light:dark period. During maintenance of the laboratory population and during the course of experiment, we used chemically untreated organic bean seeds (*Phaseolus vulgaris* c.v. “gradištanac”). To avoid any possible infestation with pests the seeds were frozen at −20 °C for 24 h prior to usage.

### 2.2. Thyme Essential Oil

Thyme oil was purchased from Sigma-Aldrich (St. Louis, MO, USA, #W306509) and its chemical composition was determined previously [71]. The major components of oil were thymol (43.52%), p-cymene (31.65%), linalool (5.38%) and carvacrol (5.11%).

### 2.3. Residual Contact Toxicity of Thyme EO on Bean Seeds

Different concentrations of thyme EO were prepared by dissolving EO in acetone. The applied concentrations for testing thyme EO toxicity on females were 0.4, 0.5, 0.6, 0.7, 0.8, 0.85, 0.9, 0.95, 1 and 1.1%, whereas in males concentrations were 0.2, 0.3, 0.4, 0.5, 0.55, 0.6, 0.65 and 0.75%. The methodology presented here has been previously used by other authors [39,64]. Bean seeds (10 g) in 90 mL glass jars were treated with 300 μL of EO solutions. Seeds with EO solutions were mixed manually for 5 min and, to evaporate the solvent, jars were left open for 20 min before adults were introduced. Accordingly, applied range of thyme EO concentrations for females and males corresponded to 120–330 and 60–225 μL of EO per kg of bean seeds, respectively. Control seeds were treated with 300 μL of acetone and were mixed in the same way as seeds treated with EO solutions. In each jar, 10 one-day-old adult females or males were introduced. Jars were covered with a piece of cloth and fixed with rubber. Five replicates per thyme EO concentration and control (acetone) were analyzed. Number of dead insects after 24 h of treatment was used to determine lethal concentrations. After that, mortality was monitored daily until all beetles died, survival time was determined and changes in mortality with advanced age were analyzed.

### 2.4. Thyme EO Effects on F1 Progeny Production

Five pairs of one-day-old bean weevils, i.e., 5 females and 5 males, were introduced into 200 mL jars with 20 g of bean seeds treated with 600 μL thyme EO solutions or 600 μL acetone (control). Seed mixing and time of solvent evaporation were the same as in the toxicity assay. Applied thyme EO concentrations were 0.1, 0.2, 0.3, 0.4, 0.5, 0.6, 0.7 and 0.8% which corresponded to the range of 30–240 μL of EO per kg of bean seeds. The insects were allowed to oviposit in the beans for 10 days. Adult emergence dynamics were monitored by daily counting of emerged adults until the last emergence of progeny. Each day adults were removed after counting. At the end of emergence period total number of adults was determined and inhibition rate (IR%) of adult emergence was calculated according to the formula:

(1) $$\mathrm{IR}\%\;=\;\frac{Nc\;-Nt}{Nc}\times 100$$

where *Nc* and *Nt* were total number of emerged adults in control and treatment jars, respectively.

### 2.5. Oviposition Deterrence

The oviposition deterrent effect of thyme EO was determined by two-choice test according to the method described by Pascual-Villalobos and Ballesta-Acosta [32] with slight modification. The test arena consisted of a large glass Petri dish (d = 15 cm) and two small Petri dishes (d = 4 cm) fixed on its opposite sides. The bottom of each small dish was covered with filter paper. On one side filter paper was treated with 100 μL of acetone and on the other side with 100 μL of thyme EO solution. The applied concentrations of EO solutions were 0.4, 0.5, 0.6, 0.7, 0.8, 0.9, 1.1, 1.3 and 1.5% which corresponded to the range of 0.4–1.5 μL of EO per dish. Petri dishes were left open to evaporate for 15 min before the start of the assay. To stimulate oviposition, one bean seed was put in each small Petri dish. In the center of test arenas, 5 females, previously kept with males for 48 h after emergence, were introduced and Petri dishes were closed. After 48 h, the number of laid eggs in control and treatment small dish was counted. For each EO concentration 7 replicates were analyzed. Oviposition deterrent index (ODI) was calculated according to the following formula:

(2) $$\mathrm{ODI}\;=\;\frac{Nt\;-\;Nc}{Nt\;+\;Nc}$$

where *Nc* and *Nt* were the numbers of laid eggs in control and treatment small Petri dishes, respectively.

### 2.6. Oxidative Stress Indices

Oxidative stress indices (carbonyl proteins, malondialdehyde (MDA) and thiol groups) were quantified in bean weevil after one-day-old females and males were exposed to different concentrations of thyme EO for 1 day. Three concentrations were chosen according to the concentration which provoked 50% mortality (LC_50_). Bean seeds were treated either with acetone (control) or with thyme EO solutions at concentrations of 1/5 LC_50_, 1/2 LC_50_ and LC_50_.

To measure oxidative stress indices, five replicate homogenates per experimental group with five beetles per replicate were prepared. Beetles were manually homogenized in 0.5 mL of 50 mM K-phosphate buffer pH 7.4 containing 1 mM EDTA and 1 mM PMSF. For determination of lipid peroxidation 0.01% BHT was also added in homogenization buffer. After sonication, homogenates were centrifuged at 10,000× *g* for 10 min at 4 °C. For carbonyl proteins quantification, supernatants were incubated with 1% streptomycin sulphate to precipitate nucleic acids which interfere with the assay, and then centrifuged at 16,000× *g* for 5 min at 4 °C. The soluble protein content was determined according to Bradford [72], with bovine serum albumin used as a standard. Multiskan Spectrum spectrophotometer (Thermo Electron Corporation, Vantaa, Finland) was used for Bradford and oxidative stress indices assays. All indices of oxidative stress were expressed as nmol per mg protein.

Carbonyl groups in oxidized proteins were quantified by simplified 2,4-dinitrophenylhydrazine (DNPH) alkaline assay [73]. After protein precipitation with equal volume of 20% TCA, protein pellets were resuspended in 50 mM K-phosphate buffer pH 7.4 containing 1 mM EDTA and kept in deep freeze at −70 °C until assay procedure. In contrast to the original method [74], DNPH derivatization of sample proteins and forming 2,4-dinitrophenylhidrazone in acid was followed by neutralization with NaOH. Adding the strong base shifted the maximum absorbance wavelength from 370 to 450 nm and eliminated the interference of unbound DNPH. Absorbance was read at 450 nm and carbonyl proteins concentration was calculated using an extinction coefficient of 22.308 mM^−1^ cm^−1^.

Lipid peroxidation was evaluated indirectly by the thiobarbituric acid reactive substances (TBARS) colorimetric assays [75]. In this procedure TBA reacts with malondialdehyde-like (MDA) products of in vivo lipid peroxidation and generates MDA-TBA adduct which is quantified at 532 nm according to the standard curve prepared by using malondialdehyde tetrabutylammonium salt (0.5–10 μM).

Total thiols (T-SH) were measured by the Ellman’s procedure [76]. Thiols in supernatants react with 5,5′-Dithiobis(2-nitrobenzoic acid) (DTNB) and yield 5-Mercapto-2-nitrobenzoic acid which absorbs at 412 nm (extinction coefficient of 14.103 mM^−1^ cm^−1^). The method of Sedlak and Lindsay [77] was used for concomitant determination of non-protein and protein bound SH groups (low-molecular mass L-SH and high-molecular mass H-SH groups, respectively). L-SH was determined in supernatants after protein precipitation with TCA while H-SH was calculated by subtracting L-SH from T-SH.

### 2.7. Statistical Methods

To estimate effective thyme EO concentrations, we applied Probit analysis [78] (PROC PROBIT, SAS Institute 2004, Cary, NC, USA). All control beetles were alive after 24 h of exposure to bean seeds treated with acetone and, thus, there was no need for correction of mortality data in the treatment groups. Significant differences between female and male low lethal and lethal concentrations were estimated according to non-overlapping confidence intervals.

Based on mortality change during lifetime of beetles, Kaplan Meier survival probability was calculated and survival analysis was performed (PROC LIFETEST, SAS Institute, 2004). The influence of thyme EO concentration on survival distribution was evaluated by log-rank test. To assess the cause of survival time change in response to EO exposure we also analyzed mortality data by using WinModest software [79]. The pattern of changes in mortality was determined by parameters *a* and *b* of the Gompertz model (*u_x_* = a*exp*^bx^*, *u_x_* is the predicted instantaneous mortality rate at age *x*). Parameter *a* is initial (baseline) age-independent mortality which reflects basal sensitivity to EO induced stress. Age-dependent parameter *b* refers to exponential increase in mortality over time which reflects the rate of increase in stress sensitivity, i.e., aging rate. The significance of differences in Gompertz parameters between control and treatment groups was evaluated by log-likelihood-ratio test.

Since data on survival time and ODI had non-homogeneous variances, we used Welch ANOVA on non-transformed data. Differences of treatment groups from the control group in survival time and ODI were revealed by Games–Howell test followed by Bonferroni correction [80]. To reveal significant differences in the number of laid eggs between control and treatment small Petri dish, ^t^-test for dependent samples was applied. To achieve normal distribution of data, log(*X* + 0.5)-transformation was used in groups with 0.7, 1.3 and 1.5% EO in treatment Petri dish.

Classic one-way ANOVA was carried out on the $\sqrt{X\;+\;0.5\;}$ transformed values of the total number of emerged adults. TableCurve 2D software (SPSS, Chicago, IL, USA) was used to fit non linear curves to daily emergence data and curve parameters were compared according to overlapping confidence intervals.

Differences in oxidative stress indices were analyzed by two-way ANOVA to evaluate significance of main (thyme oil concentration and sex) and interaction effects on the levels of carbonyl proteins, MDA and thiol groups. ANOVAs were performed on log-transformed values of L-SH and non-transformed values of other oxidative stress indices. Specific comparisons among experimental groups were done by Duncan’s test while differences between control and treatment groups were tested by Dunnett test.

## 3. Results

### 3.1. Residual Contact Toxicity of Thyme EO

**Figure 1:**
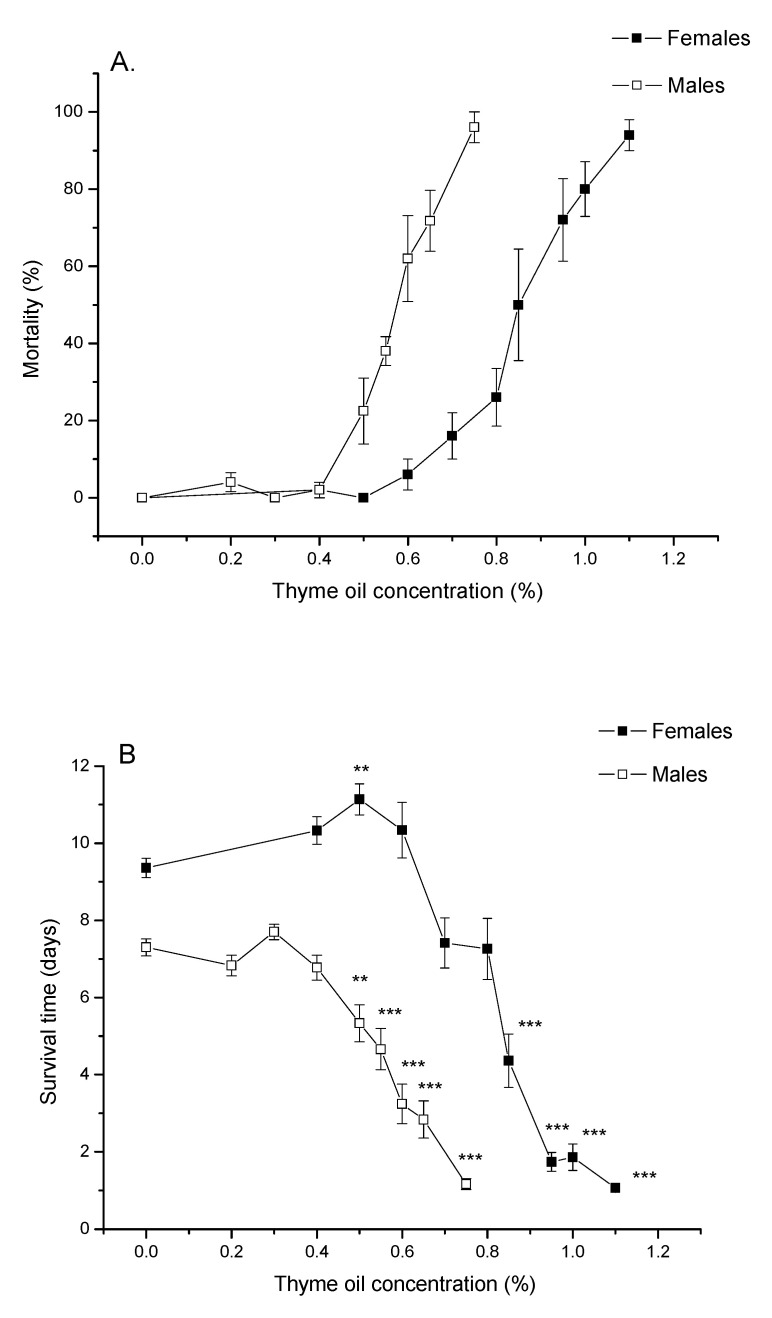
(**A**) Percentage of mortality after 24 h of exposure to thyme oil (means and standard errors for 5 replicates) and (**B**) survival time in *Acanthoscelides obtectus* females and males (means and standard errors for beetles that survived the 24 h essential oil (EO) treatment). Significant differences between control and EO-treated groups within each sex are presented with asterisks (** *p* < 0.01, *** *p* < 0.001).

Figure 1A demonstrates that percentage of *A. obtectus* mortality after 24 h of exposure to bean seeds treated with thyme EO increased with increasing EO concentration both in females and males. Pearson’s goodness-of-fit test confirmed that concentration–mortality response fitted the probit distribution (Table 1). Low lethal (LC_30_) and lethal concentrations (LC_50_ and LC_99_) significantly differed between females and males (non-overlapping confidence intervals in Table 1). Females were more resistant since they started to die at higher concentrations (Figure 1A) and higher EO concentrations were needed to provoke the same mortality as in males (Table 1). 

### 3.2. Thyme EO Impact on Survival Time

Increasing thyme EO concentrations led to shorter lives of females (Welch ANOVA, F_9,180.68_ = 283.51, *p* < 0.0001) and males (F_8,178.27_ = 133.34, *p* < 0.0001). At EO concentration of 0.5% survival time of females was significantly increased compared to the control group (*p* = 0.0030, Games–Howell test with Bonferroni correction) while significant reduction of survival times can be noticed at concentrations ≥0.85% in females and concentrations ≥0.5% in males (Figure 1B).

**Figure 2:**
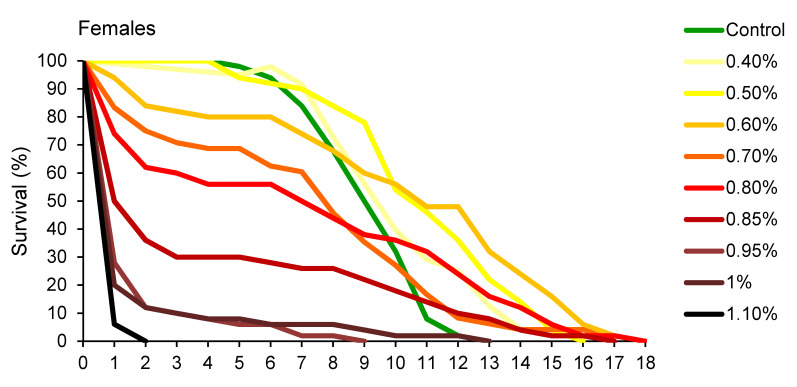

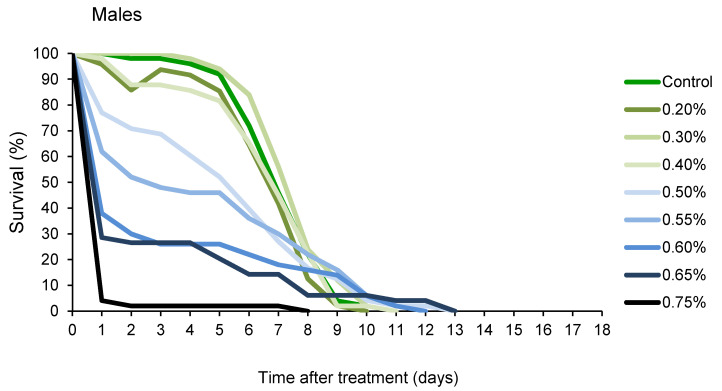
Changes in percentage of survival over time for *Acanthoscelides obtectus* females and males exposed to different concentrations of thyme oil.

Changes in percentage of survival over time, presented in Figure 2, revealed that *A. obtectus* adults exposed to EO started to die earlier, compared to the control group. Survival distribution was significantly affected by thyme oil concentration both in females (χ^2^ = 323.45, df = 9, *p* < 0.0001) and males (χ^2^ = 131.83, df = 8, *p* < 0.0001). Results on the values of Gompertz parameters presented in Table 2 showed that higher initial mortality (*a*) was related to lower exponential increase in mortality (*b*). Concentrations below 0.5% did not affect Gompertz parameters *a* and *b* in males while in females exposed to concentrations of 0.8% and lower, increased initial mortality (*a*) was compensated for by retarded aging (reduced parameter *b*) (Table 2). The only exception in females was 0.5% EO concentration that by reducing aging rate (*b*) without change in initial mortality (*a*), led to higher survival time in treated compared to control beetles (Figure 1B, Table 2).

### 3.3. Thyme EO Impact on F1 Progeny Number

**Figure 3:**
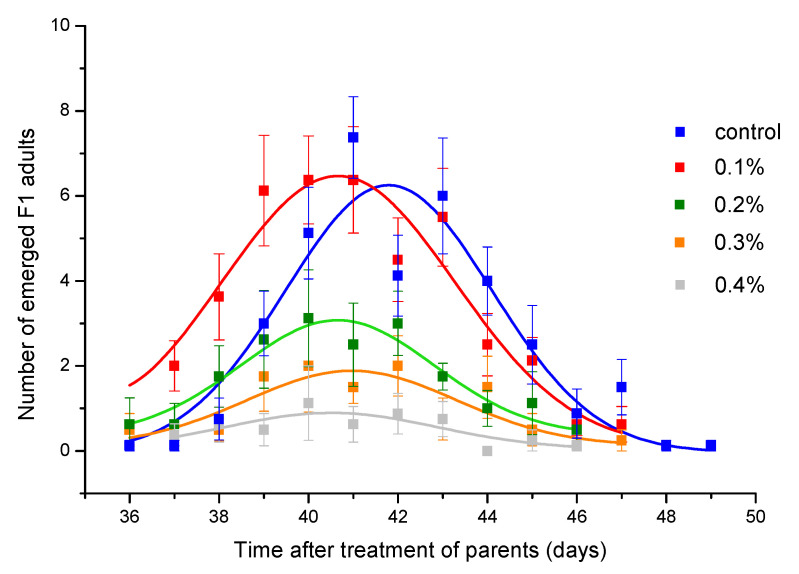
Daily emergence of *Acanthoscelides obtectus* F1 adults. Symbols represent means and standard errors for 8 replicates. Curve fit lines are from the equation in Table 4.

Toxicity of thyme EO was also reflected in reducing the F1 progeny number (Table 3). Although concentration of 0.2% caused mortality lower than 5% (Figure 1B), the number of emerged adults was significantly reduced. At concentration of 0.6% which did not affect female survival, but reduced male survival by about 50%, progeny production was almost completely inhibited. The concentration that led to 50% inhibition rate of adult emergence was estimated to be 0.219% (CI = 0.194, 0.240%). Daily emergence curves presented in Figure 3 show that peak of emergence decreases with increase in EO concentration. The peak was significantly reduced compared to control group if parental generation was exposed to thyme EO at concentrations ≥0.2% (Table 4). However, the EO concentration did not affect the curve position (parameter *b* in Table 4) as well as curve width (parameter *c* in Table 4).

### 3.4. Oviposition Deterrent Activity of Thyme EO

Results of the two-choice test presented in Table 5 clearly demonstrate that oviposition deterrence increased with thyme EO concentration. Significant differences between the numbers of eggs laid in the control and treatment dish were recorded at concentrations ≥0.6%. Oviposition deterrent index was significantly affected by EO concentration (Welch ANOVA, F_8,22.27_ = 8.85, *p* < 0.0001). Compared to the control group where both small Petri dishes in two-choice test arena contained acetone, significantly more negative ODI was recorded when high concentration of EO solution (≥0.9%) was applied in a small treatment dish (Table 5). Effective concentrations of thyme EO, which provoked 50% deterrence (corresponds to ODI = −0.5), was calculated to be 0.719% (CI = 0.640, 0.800%).

### 3.5. Thyme EO Effects on Oxidative Damage and Thiol Content

**Figure 4:**
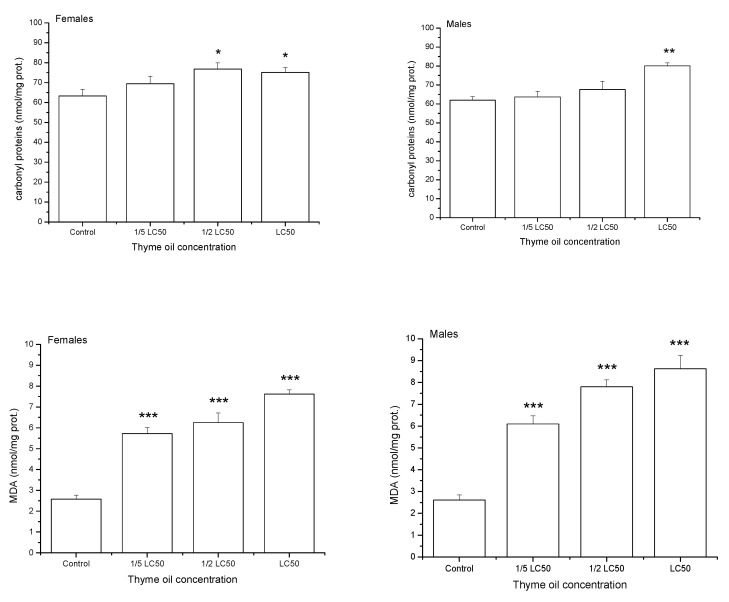
Carbonyl proteins and malondialdehyde (MDA) contents (means and standard errors for 5 replicates) in *Acanthoscelides obtectus* adults one day after exposure to bean seeds treated with thyme oil. Significant differences in treatment groups from the control are marked by asterisks (* *p* < 0.5, ** *p* < 0.01, *** *p* < 0.001).

After one-day exposure to thyme EO, the levels of oxidatively damaged proteins (carbonyl proteins-CP) and lipids (MDA) gradually increased with EO concentration (Figure 4). Results of two-way ANOVA revealed a significant effect of EO concentration on CP (F_3,32_ = 9.23, *p* = 0.0002) and MDA (F_3,32_ = 87.74, *p* < 0.0001). On average, sexes did not differ in the CP level (F_3,32_ = 8.49, *p* = 0.0065), whereas males had more damaged lipids than females (F_3,32_ = 1.69, *p* = 0.2030). Changes in indices of oxidative damage with concentration were similar between sexes both for CP (non-significant concentration × sex interaction effect in two-way ANOVA, F_3,32_ = 1.99, *p* = 0.1346) and MDA (F_3,32_ = 1.76, *p* = 0.1750).

**Figure 5:**
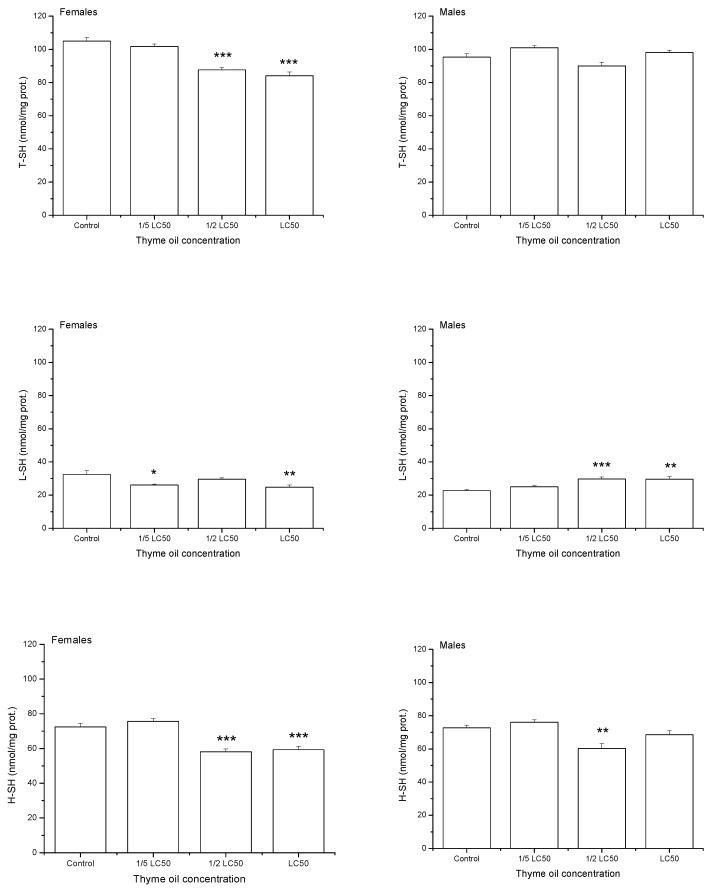
Total thiols (T-SH), low-molecular mass thiols (L-SH) and high-molecular mass thiols (H-SH) contents (means and standard errors for 5 replicates) in female and male *Acanthoscelides obtectus* adults one day after exposure to bean seeds treated with thyme oil. Significant differences in treatment groups from the control are marked by asterisks (* *p* < 0.5, ** *p* < 0.01, *** *p* < 0.001).

On average, one-day exposure to increasing thyme EO concentrations reduced levels of total thiols (T-SH) and high-molecular mass thiols (H-SH) (T-SH: F_3,32_ = 25.33, *p* < 0.0001, H-SH: F_3,32_ = 29.09, *p* < 0.0001, Figure 5). Low-molecular mass thiols (L-SH) were also significantly influenced by thyme EO concentration (F_3,32_ = 4.08, *p* = 0.0146) but changes in their level were less expressed. On average, the highest L-SH value was recorded in 1/2 LC_50_ group, even higher than in the control one (Duncan post hoc test, *p* = 0.0413), 1/5 LC_50_(*p* = 0.0029) and LC_50_ group (*p* = 0.0430). T-SH level did not differ between the sexes (F_1,32_ = 1.47, *p* = 0.2336) but females had less H-SH (F_1,32_ = 4.54, *p* = 0.0410) and slightly more L-SH than males (F_1,32_ = 3.32, *p* = 0.0776). Differences in H-SH level between females and males were marginally dependent on EO concentration (F_3,32_ = 2.27, *p* = 0.0999) and significant difference was recorded only at the highest examined concentration, where males had more H-SH than females (Duncan post hoc test, *p* = 0.0035). Concentration × sex interaction was significant for T-SH (F_3,32_ = 15.11, *p* < 0.0001) and L-SH (F_3,32_ = 13.32, *p* < 0.0001). While females exposed to acetone showed higher levels of T-SH (*p* = 0.0013) and L-SH (*p* < 0.0001) than males, the opposite trend was noticed when insects were exposed to LC_50_ thyme EO (*p* < 0.0001, *p* = 0.0108 for T-SH and L-SH, respectively).

## 4. Discussion

Thyme and thyme essential oil have been traditionally used as tea and spice in human nutrition, for food preservation and various medical treatments [81]. EPA and FDA indicated their safe use in food products because of low risks for the environment and human health [82]. Many studies on storage and other pest insects recorded significant influence of thyme EO on pest survival and behavior [83,84,85,86,87,88,89,90]. Lamiaceae EOs applied as fumigants have been extensively tested for the activity against *A. obtectus* [61,62,91,92,93] and several recent studies dealt with the effects of their application on bean seeds [63,67,71]. Here we evaluated efficacy of thymol chemotype of the thyme oil as residual contact insecticide and oviposition deterrent against *A. obtectus*.

Our study demonstrated concentration-dependence of thyme EO effects on *A. obtectus* mortality, survival time and shape of survival curves. Insecticidal efficacy of thyme EO was similar to the effects of some commercial botanical insecticides [94], but lower compared to conventional insecticides [95]. The main compounds of the thyme oil are oxygenated monoterpens thymol, carvacrol, and linalool, and non-oxygenated monoterpene p-cymene [71]. They are known for strong fumigant toxicity against *A. obtectus* and adult longevity reduction [70]. Additionally, contact application of thymol and linalool on glass [71] and essential oils of *Clausena anisata* (Willd.) J.D. Hook ex. Benth. (Rutaceae) [68], *Syzygium aromaticum* L., and *Cinnamomum zeylanicum* L. on bean seeds [64] induce high mortality of *A. obtectus* adults.

Concentration-dependent decrease in survival time of *A. obtectus* at higher EO concentrations stem from increased initial mortality (Gompertz parameter *a*). Initial mortality depends on capability of an organism to adequately respond to stress challenges that disturb organism homeostasis. Many stressors including EOs, increase the production of reactive oxygen species. At low EO concentrations various adaptive responses are induced while at high EO concentrations, stress intensity exceeds a certain threshold and these responses are not sufficient to remove ROS leading to macromolecular damage [52,96,97]. We suggest that beetles which survived after 24 h of EO treatment initially die due to delayed effects of the EO compounds on physiological systems. Rapid insecticidal activity suggests involvement of neurological mechanisms of toxicity. Thymol, a major component of thyme EO is known as inhibitor of acethylcholine esterase in insects [52,98]. Increased oxidative damage to macromolecules may contribute to high baseline mortality whereas induction of antioxidative responses slows down the rate of aging (decreased Gompertz parameter *b*). For instance, Shahriari et al. [53] recorded elevated activity of superoxide dismutase, peroxidase, catalase, ascorbate peroxidase and glutathione S transferase in a storage pest *Ephestia kuehniella* Zeller in response to the presence of thymol in food. Despite induction of these defense mechanisms, the level of damaged lipids was increased and glutathione, a low-molecular mass thiol, was more oxidized.

Numerous studies confirmed that EOs and their components exert significant influence on insect metabolic pathways, elevate ROS production as a consequence of impairment of mitochondrial function, provoke damage to lipids, proteins and DNA, lead to cell membrane disruption, deplete reduced thiols, and induce activities of antioxidative enzymes and synthesis of Hsp [96,97,99,100,101,102]. Our results on the gradual elevation of oxidatively damaged proteins and lipids, and reduced level of thiols from sublethal (1/5 LC_50_, 1/2 LC_50_) to lethal EO concentrations (LC_50_) after 24 h of exposure showed that oxidative stress play an important role in thyme EO toxicity against *A. obtectus*. Another finding of our study refers to the higher tolerance of *A. obtectus* females to thyme EO treatments compared to males. Namely, lethal concentrations of EO were higher and survival time was longer which is in accordance with previously described higher oxidative stress resistance of females [103,104] as well as with the present results on the lower level of oxidatively damaged lipids and marginally higher level of low-molecular mass thiols. Similar to our results, *A. obtectus* females were more tolerant to fumigant action of four major components of thyme oil [70], and thyme, lavender, rosemary, mint and other EOs [61,69,105]. Further investigations are needed to fully understand underlying mechanisms of thyme oil toxicity and its sexual dimorphism.

Sublethal concentrations of essential oils may also exhibit significant beneficial or detrimental influence on pest life history components, behavior and physiology [30]. For example, we showed beneficial influence of sublethal thyme oil concentration (0.5%) on female survival time. Similarly, in *Ceratitis capitata*, low concentration of monoterpene limonene prolonged lifespan and increased fecundity [106]. Haddi et al. [44] found elevated number of *Sitophilus zeamais* larvae per maize grains when parents were exposed to low concentration of *S. aromaticum* and *C. zeylanicum* EOs for 24 h. This stimulatory effect of low EO concentration (hormesis) was accompanied with decreased locomotion and decreased respiration rate of insects. The authors suggested involvement of endocrine, antioxidant and detoxification systems in regulation of EO-induced hormesis and emphasize the importance of choice of proper EO concentrations that would not disturb pest control efficacy or contribute to evolution of pest resistance.

Detrimental influence of sublethal and low lethal thyme oil concentrations on adult emergence recorded in our study can be explained by decrease in male survival time, oviposition deterrent effects in females and impairment in oxidative stress indices. No-choice tests revealed that thyme oil reduced fecundity of *A. obtectus* females after contact [71] and fumigant application which also affected larval penetration into seeds [69]. Choice tests revealed good repellent, and feeding deterrent activity of thyme oil in many pests [84,86,88,107]. EOs may also change adult emergence by affecting survival at preadult developmental stages of *A. obtectus* [61,92]. Further studies are needed to understand mechanisms of adult emergence reduction in thyme EO exposed *A. obtectus* at the level of mating, oviposition patterns, and larval vitality and penetration ability. In addition to total adult emergence, exposure to EOs can change the shape of daily emergence curve. Low lethal and lethal concentrations of *Syzygium aromaticum* and *Cinnamomum zeylanicum* EOs on seeds delayed emergence of *A. obtectus* [64] and *Callosobruchus macullatus* adults [46]. In contrast, our results showed that thyme EO concentrations did not induce significant changes in the emergence patterns.

## 5. Conclusions

Thymol chemotype of thyme oil applied on bean seeds significantly reduced survival of *A. obtectus* adults in a concentration-dependent manner. Decline in survival time of both females and males was a consequence of elevated initial mortality which can be explained by increased oxidative damage of proteins and lipids, and decreased level of thiols. Females who contained less oxidatively damaged lipids and more thiols were more tolerant to thyme EO treatment. Sublethal concentrations exerted significant influence on oviposition deterrent index, biochemical parameters and adult emergence. The EO concentrations with no effect on *A. obtects* survival provoked up to 85% inhibition of adult emergence. Efficacy of such low EO concentrations in suppression of adult emergence implies that thyme EO might be a cost-effective and environmentally low risk botanical insecticide for bean protection against *A. obtectus*.

## Figures and Tables

**Table 1:** Residual contact toxicity of thyme oil against *Acanthoscelides obtectus* females and males.

Sex	Slope ± SE (CI)	LC_30_^a^ (CI)	LC_50_^a^ (CI)	LC_99_^a^ (CI)	χ^2^	*p*
Females	12.00 ± 1.14 (9.76; 14.23)	0.769 (0.738; 0.794)	0.850 (0.825; 0.876)	1.329 (1.234; 1.476)	2.67	0.7505
Males	13.46 ± 1.45 (10.62; 14.16.30)	0.524 (0.503; 0.542)	0.574 (0.556; 0.591)	0.854 (0.794; 0.953)	0.82	0.9361

^a^ Lethal concentrations are presented with 95% confidence intervals (CI) and expressed in %. χ^2^, *p*—results of Pearson’s goodness-of-fit test (df = 5 for females and df = 4 for males).

**Table 2:** Effects of different concentrations of thyme essential oil (EO) on Gompertz mortality parameters *a* and *b*. Baseline mortality (*a*) and exponential increase in mortality with age (*b*) are presented with 95% confidence intervals (CI). Significant differences from the control group are marked with asterisks (log-likelihood ratio test, df = 1, *p* < 0.05).

Thyme EO Concentration (%)	*a* (×10^−3^)	(CI)	*b*	(CI)
Females				
0	0.95	(0.25, 3.62)	0.64	(0.51, 0.79)
0.4	4.79 *	(1.88, 12.20)	0.38 *	(0.30, 0.48)
0.5	2.86	(1.00, 8.18)	0.40 *	(0.31, 0.50)
0.60	16.15 *	(7.48, 34.86)	0.21 *	(0.15, 0.30)
0.70	50.41 *	(27.72, 91.67)	0.16 *	(0.10, 0.25)
0.80	79.22 *	(45.65, 137.47)	0.09 *	(0.04, 0.18)
Males				
0	2.75	(0.91, 8.29)	0.69	(0.56, 0.85)
0.2	3.03	(0.92, 9.95)	0.72	(0.57, 0.91)
0.3	1.69	(0.51, 5.64)	0.72	(0.58, 0.89)
0.40	7.53	(2.83, 20.06)	0.56	(0.43, 0.71)
0.50	75.69 *	(42.18, 135.83)	0.20 *	(0.13, 0.33)
0.55	142.49 *	(84.30, 240.83)	0.10 *	(0.04, 0.26)

**Table 3:** Number of emerged *Acanthoscelides obtectus* adults in F1 generation (means and standard errors for 8 replicates) whose parents were exposed to different concentrations of thyme essential oil (EO).

Thyme EO Concentration (%)	Number of Emerged Adults		Inhibition of Adult Emergence (%)
Control	35.50 ± 3.87	a	0
0.1	40.38 ± 6.60	a	−13.73
0.2	18.38 ± 5.32	b, *	48.24
0.3	12.00 ± 3.46	bc, **	66.20
0.4	5.25 ± 3.17	cd, ***	85.21
0.5	2.75 ± 2.75	d, ***	92.25
0.6	1.71 ± 1.71	d, ***	95.17
0.7	0		100
0.8	0		100
ANOVA	F_6,48_ = 15.02, *p* < 0.0001		

F, *p*—results of one-way ANOVA on $\sqrt{X\;+\;0.5\;}$ transformed values of the number of emerged adults. a,b,c,d mark significant differences among experimental groups (Duncan’s post hoc test, *p* < 0.05). * *p* < 0.05, ** *p* < 0.01, *** *p* < 0.001 mark significant differences from the control group (Dunnett’s test).

**Table 4:** Parameters of non-linear regressions (means and confidence intervals in parentheses) of daily adult emergence in F1 generation depending on thyme oil concentration (C) according to the model *y* = a*exp(−0.5*((*x* − *b*)/*c*)^2^) + *d*.

C (%)	*a*	*b*	*c*	*d*	df_error_	F	*p*	R^2^
0	6.29 (5.08, 7.49)	41.79 (41.39, 42.19)	2.30 (1.67, 2.94)	−0.03 (−1.06, 0.99)	108	37.36	<0.0001	0.51
0.1	6.38 (4.16, 8.59)	40.66 (40.15, 41.18)	2.61 (1.45, 3.77)	0.09 (−2.21, 2.40)	84	18.86	<0.0001	0.40
0.2	2.70 * (1.09, 4.30)	40.65 (39.80, 41.49)	2.16 (0.33, 3.99)	0.38 (−1.28, 2.04)	84	5.53	0.0016	0.16
0.3	1.76 * (0.64, 2.88)	40.96 (39.95, 41.98)	2.35 (0.22, 4.47)	0.13 (−1.02, 1.27)	92	4.58	0.0049	0.13
0.4	0.85 * (−0.32, 2.03)	40.51 (38.84, 42.17)	2.37 (1.69, 6.44)	0.04 (−1.20, 1.29)	76	1.39	0.2525	0.05

* Significant differences between control and treatment groups.

**Table 5:** Number of eggs laid in control dish and treatment Petri dish and oviposition deterrence index (ODI) (means and standard errors for 7 replicates) depending on thyme oil concentration (C). Significant differences are given in bold.

C (%)	Control		Treatment		t	*p*	ODI		F	*p*
0	27.71	4.26	25.14	2.56	0.46	0.6623	−0.022	0.112		
0.4	26.71	4.87	22.71	4.86	0.55	0.5489	−0.101	0.136	0.20	1.0000
0.5	31.43	7.10	18.43	5.10	1.24	0.2616	−0.215	0.217	0.62	1.0000
0.6	39.57	8.03	15.00	4.64	3.15	**0.0197**	−0.457	0.137	6.02	0.2491
0.7	28.43	5.13	12.57	6.87	2.69	**0.0359**	−0.579	0.223	4.98	0.4245
0.9	35.00	3.51	8.86	2.51	12.96	**0.0000**	−0.643	0.067	22.59	**0.0066**
1.1	36.29	5.42	7.86	2.24	6.51	**0.0006**	−0.685	0.082	22.79	**0.0046**
1.3	38.57	5.92	2.57	1.41	5.73	**0.0012**	−0.858	0.074	38.77	**0.0007**
1.5	31.57	3.29	1.00	0.69	7.89	**0.0002**	−0.924	0.051	53.55	**0.0005**

t, *p*—results of t-test for dependent samples. F, *p*—results of Games–Howell test with Bonferroni correction.
